# Reducing the extent of facetectomy may decrease morbidity in failed back surgery syndrome

**DOI:** 10.1186/s12891-019-2751-5

**Published:** 2019-08-09

**Authors:** Jingchi Li, Xiaoyu Zhang, Wenqiang Xu, Zhipeng Xi, Lin Xie

**Affiliations:** 1 Department of Orthopedic Surgery, Jiangsu Province Hospital on Integration of Chinese and Western Medicine, 100th .Shizi Street , Nanjing, 210028 Jiangsu Province, People’s Republic of China; 2 Department of Spine Surgery, Third Clinical Medical College of Nanjing University of Chinese Medicine, Nanjing, 210028 Jiangsu China

**Keywords:** Percutaneous transforaminal endoscopic discectomy, Facetectomy, Failed back surgery syndrome, Finite element research

## Abstract

**Background:**

Percutaneous transforaminal endoscopic discectomy (PTED) is widely used for the treatment of lumbar disc herniation. Facetectomy in PTED is necessary for accessing the intraspinal region and for decompressing the exiting nerve roots in patients who suffer from hypertrophy of the facet joints. However, this may increase morbidity in failed back surgery syndrome (FBSS) and has not been clearly elucidated.

**Methods:**

A three-dimensional lumbosacral model was reconstructed and validated. And corresponding models after PTED with one-quarter and one-half excisions of the superior articular process were reconstructed. The maximum shear stress on the annulus in L5, von Mises stress of the facet cartilage, maximum principle capsular strain and deformation of the lumbosacral model were calculated using finite element methods.

**Results:**

Calculated results show no significant differences in the complete model and the model with one-quarter excision of the superior articular process, but all biomechanical indexes have been deteriorated under most of the loading conditions tested in the model with one-half excision of the superior articular process.

**Conclusions:**

Less facetectomy is better because it may reduce the risk of biomechanical deterioration and consequently, that of FBSS.

## Background

Failed back surgery syndrome (FBSS) is a frequently occurring postoperative complication [1]. Many studies have reported that biomechanical deterioration is the most crucial reason for postoperative complications such as FBSS [2–6].

**Fig. 1:**
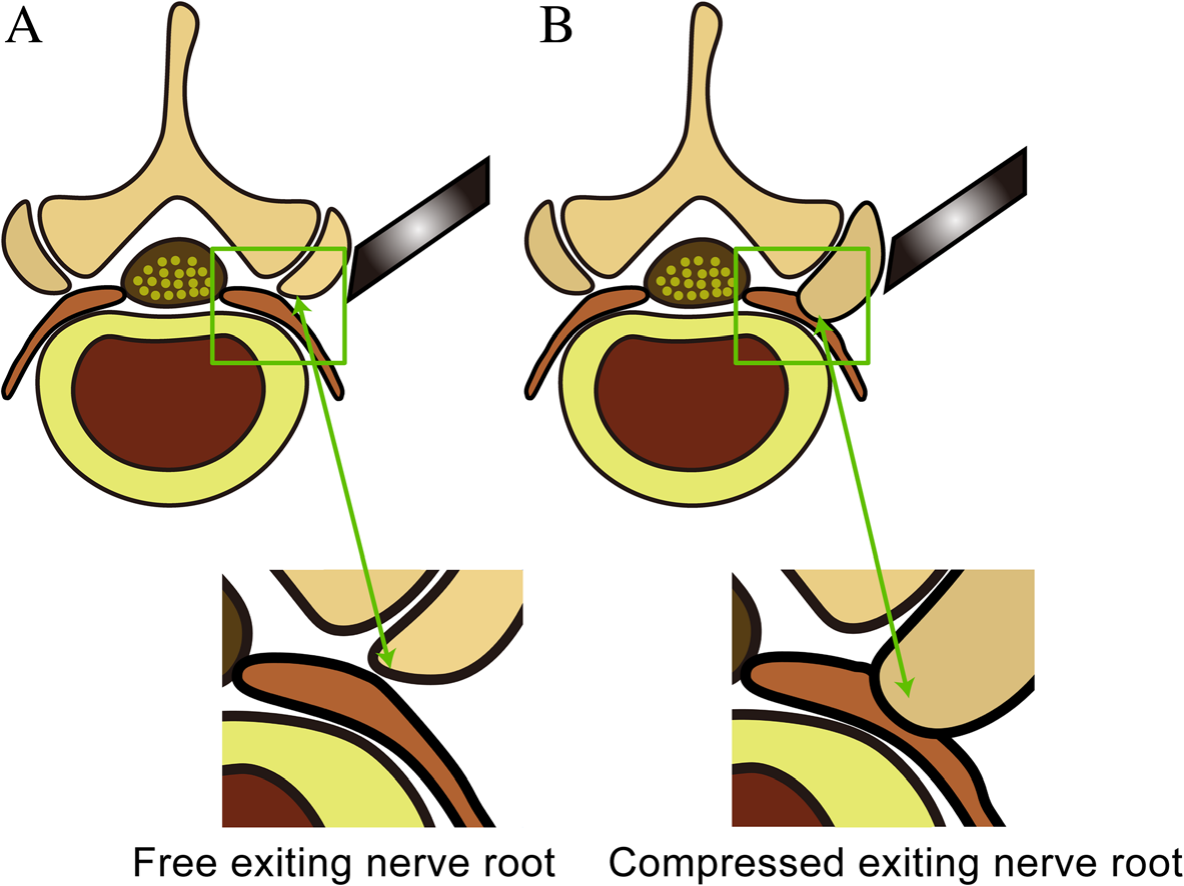
The necessity of facetectomy in patients suffer from exiting nerve roots compression. **a**. Free exiting nerve root. **b**. Compressed exiting nerve root

Percutaneous transforaminal endoscopic discectomy (PTED) has already been used in the treatment of lumbar disc herniation (LDH) [7, 8]. In PTED, it is necessary to perform facetectomy to expand the neuroforamen. And in patients who suffer from hypertrophy of the facet joints, extensive facetectomy is needed for the decompression of exiting nerve roots. However, such a procedure may be closely associated with postoperative complications (Fig.1).

Aetiologies related to facet joints involved in the pathogenesis of low back pain (LBP) occur in 15–37% of patients after surgery [9, 10], .and injury to the facet joint has been reported to cause a series of postoperative complications [6, 11]. Meanwhile, tears in the posterior annulus is an important trigger for discogenic LBP and LDH, which are two principal causes of FBSS [1, 12, 13]. The facet cartilage plays a key role in protecting the posterior annulus during torsion, and the capsule of the facet joint plays a similar role during flexion [14, 15]. Given that facetectomy does not directly cause injury to the articular surface and the capsule, variation in the stress distribution may result in secondary damage and increase the incidence of annulus tears [1, 12]. Furthermore, rich innervation is a typical histological feature of the facet capsule, and any increase in the mobility of the facet joint will affect the strain on the capsule and stimulate the nociceptors, thereby leading to LBP [16, 17, 18]. In addition, surgical intervention will change the force applied on the facet cartilage, leading to possible degeneration [19, 20]. Besides, the facet joint is important in the maintenance of spine stability [16, 21], and iatrogenic instability is common in patients suffering from FBSS [22, 23]. Taken together, we hypothesise that facetectomy and extensive facetectomy increase the risk of FBSS. However, this conjecture lacks the theoretical basis of biomechanics.

As a mechanical simulation research method, finite element analysis has been widely used in the investigation of postoperative biomechanical variations to infer the risk of postoperative complications [3–5, 24]. To clarify our hypothesis and provide theoretical guidance for the PTED application, we reconstructed a lumbosacral finite element model (FEM) to determine whether varying extents of facetectomy will affect its biomechanical indexes. To the best of our knowledge, published literatures have not adequately clarified this issue.

## Method

### L3-S1 FEM of a healthy spine

A three-dimensional model from L3 to S1 was reconstructed based on high-resolution computed tomography (CT) image of a 24-year-old male volunteer who was one of the authors of this manscript and without any history of lumbar diseases. The model comprised four vertebral bodies, three segments of intervertebral discs (IVDs), six facet joints and six ligaments. Components that could not be clearly distinguished by CT were reconstructed based on anatomical observations [4, 24].

The bone structure included a cortical bone shell (0.8 mm), a cancellous bone core, two endplates (0.8 mm) and posterior structure. The IVD consisted of the inner nucleus and the surrounding annulus, and the nucleus occupied 44% of the cross-sectional area of the IVD and was located slightly posterior to the centre of the disc [25, 26]. Facet joints consisted of the surrounding capsule and two cartilage surfaces (0.25 mm) [4, 5]. Ligaments, which include the anterior longitudinal ligament, posterior longitudinal ligament, ligamentum flavum (LF), intertransverse ligament, interspinous ligament and supraspinous ligament, were constructed in the preprocessing process of the finite element analysis, and the capsule of the facet joints was reconstructed similarly [3, 27].

### Spine models after PTED

**Fig. 2:**
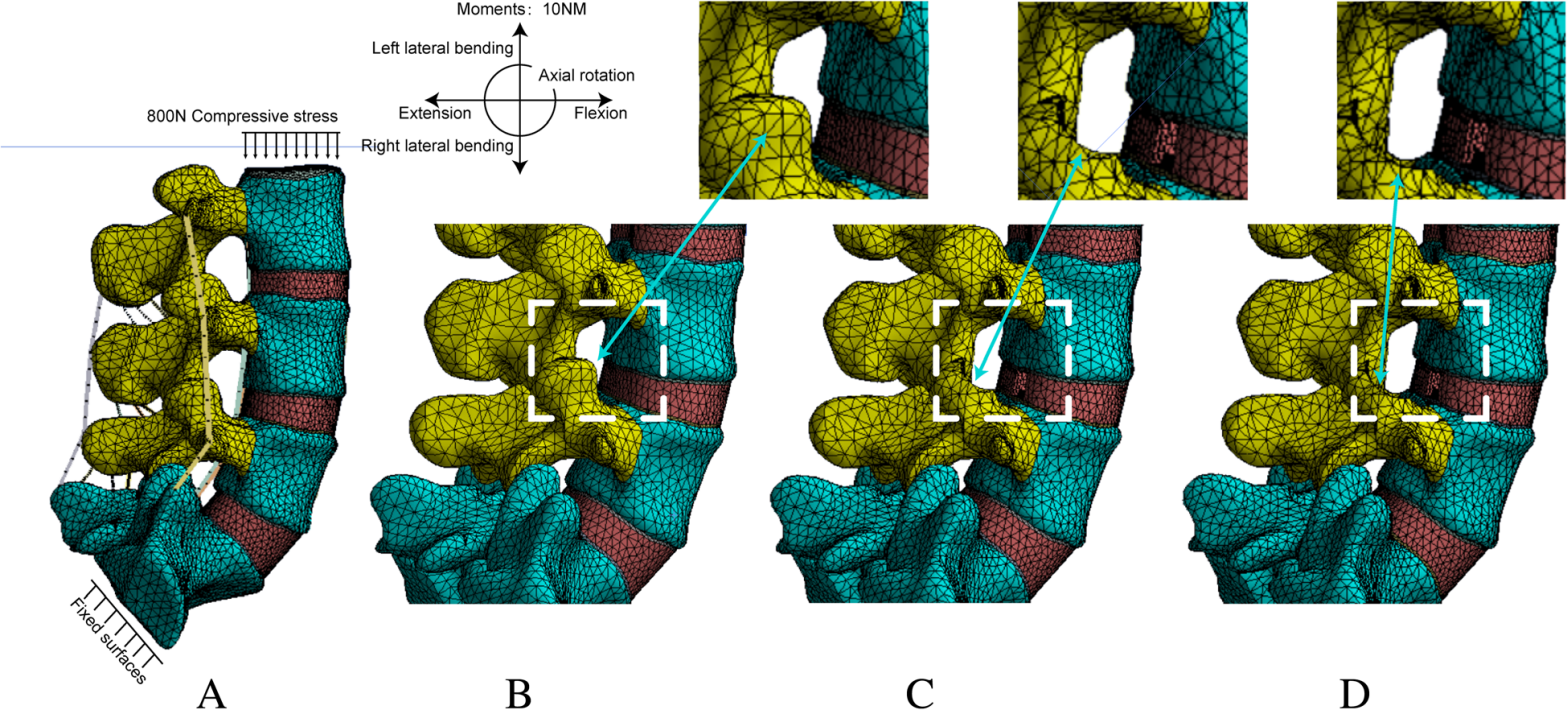
Models in the current research (ligaments have been hided for the sake of brevity in the schematic diagram of PTED). **a**. The complete lumbo-sacral model. **b**. Model1: The preoperative model. **c**. Model2: The model after one-quarter excision of the superior articular process. **d**. Model3: The model after one-half excision of the superior articular process

**Fig. 3:**
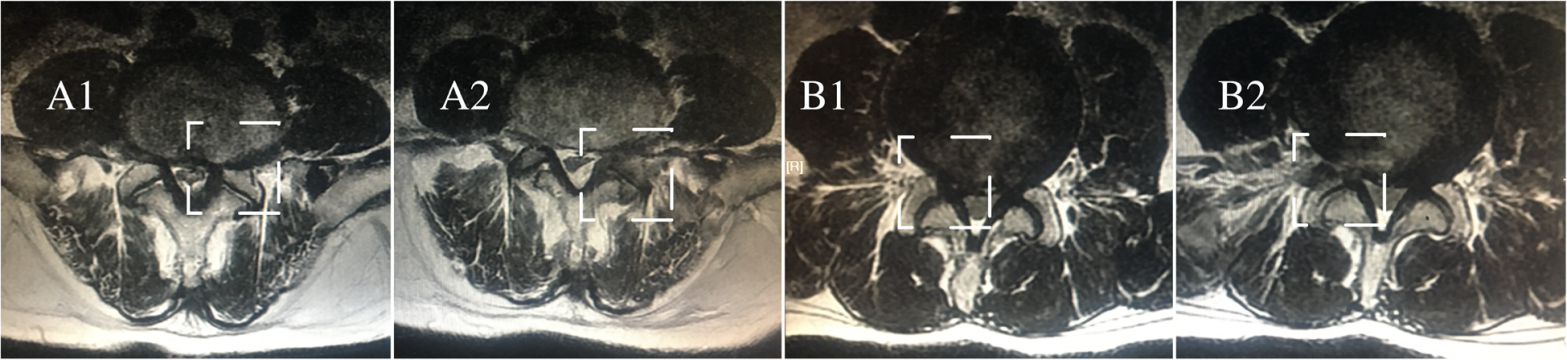
The magnetic resonance imaging (MRI) of patients subjected to PTED with different grades of facetectomy. **a**. PTED in the left side. **b**. PTED in the right side. 1. Preoperative data. 2 .Postoperative data

The reconstruction PTED model was based on the complete model described above, and the L4–L5 segment was selected for simulation due to the high incidence rate of LDH in this segment [28, 29]. Surgery was simulated on the right side; a 5-mm incision in the annulus was made to simulate the annulus tear and the nucleus was removed to imitate discectomy [7, 8]. Additionally, one-third of the LF on the right side was excised in the two PTED models. All procedures were identical except for whether they were one-quarter or one-half facetectomy procedures of the superior articular process [5, 7]. Pictorial representation of the simulated PTED procedures are shown in Fig. 2, and magnetic resonance images of patients subjected to PTED in different sides with various grades of facetectomy are shown in Fig. 3.

### Boundary and loading conditions

Boundary and loading conditions were identical in all models. All degrees of freedom were fixed below the inferior surface of S1, and five different loading conditions including flexion, extension, left lateral bending, right lateral bending and axial rotation were simulated by applying 10 NM moments on the superior surface of the L3. All simulation conditions were based on 800 N of vertical compression [25]. In addition, 85% of the force was applied on the anterior and the middle columns, while l5% was applied on the posterior column [30, 31]. For brevity, the complete model was labelled model 1, that after one-quarter excision of the superior articular process as model 2 and that after one-half excision of the superior articular process as model 3.

The tetrahedral solid elements was selected for this models as it is appropriate for filling models with complex surfaces, and we used mesh refinement in areas where mesh distortion was needed to improve mesh quality and reduce calculation error. The bounded contact type was selected for all surfaces in the lumbosacral model, as the surfaces do not separate under normal stress conditions, except at the facet cartilage surfaces, which were therefore defined as frictionless [25, 27]. Additionally, the centrum, endplates and facet cartilages were defined as being composed of isotropic and homogeneous elastic materials, the nucleus was defined as an ‘incompressible fluid bag’, the annulus as a hyper-elastic ‘Mooney–Rivlin’ material and the ligaments as tension-only cable elements. Material properties in the current model were based on published FEM studies (Tables 1 and 2) [32, 33].

**Table 1:** The material properties used in finite element models

Components	Young’s modulus (Mpa)	Poisson’s ratio	Cross-section area(mm^2)^
Cortical	12,000	0.3	/
Cancellous	100	0.2	/
Posterior elements	3500	0.25	/
Endplate	1000	0.4	/
Cartilage	10	0.4	/
Capsular	26	0.3	67.5
ALL	20	0.3	60
PLL	70	0.3	21
LF	50	0.3	60
ITL	50	0.3	10
ISL	28	0.3	40
SSL	28	0.3	30

Note: ALL: anterior longitudinal ligament; PLL:posterior longitudinal ligament; LF:ligamantum flavum; ISL:interspinous ligament; SSL: supraspinal ligament; ITL:intertransverse ligament

**Table 2:** The material properties of Intervertebral discs

Annulus		Nucleus	
C1 (MPA)	C2 (MPA)	Young’s modulus (Mpa)	Poisson’s ratio
0.2	0.05	1	0.499

## Results

### Validation of model reconstruction

**Fig. 4:**
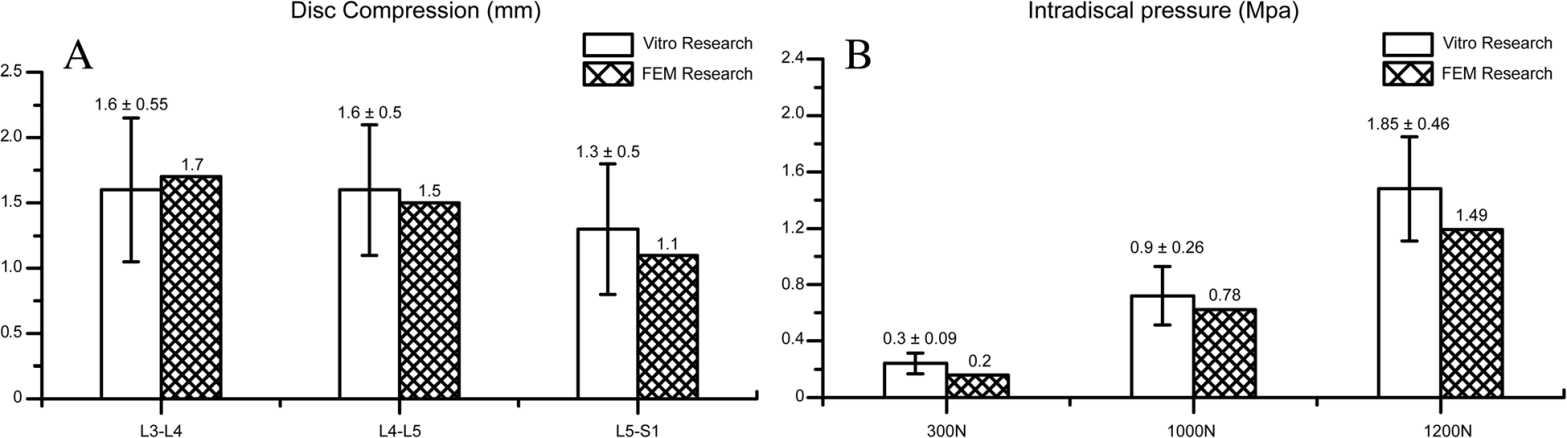
Validation process for the current model. **a**. Comparasion of the intradiscal pressure with the validated vitro study. **b**. Comparasion of the disc compression value with the validated vitro study

We validated our complete model (model 1) by comparing disc compression values and intradiscal pressure in model 1 with those from two well-validated and repeatedly cited in vitro biomechanical researches, henceforth referred to as ‘specimen’. Values of disc compression under a load of 1200 N in the specimen were 1.6 ± 0.55, 1.6 ± 0.5 and 1.3 ± 0.5 mm in L3–L4, L4–L5 and L5–S1 IVD segments [34], and values of intradiscal pressure were 0.3 ± 0.09, 0.9 ± 0.26 and 1.85 ± 0.46 MPa under 300 N, 1000 N and 2000 N loads, respectively [35]. The disc compression values of our current model were 1.7, 1.5 and 1.1 mm, and the intradiscal pressures were 0.2, 0.78 and 1.49 MPa, respectively. Significantly, values from model 1 were within one standard deviation of those reported for the specimen (Fig. 4), except for the intradiscal pressure under 300 N. Considering that it was only 0.01 MPa below the standard deviation and equal to the lowest value in the original data [35], the complete model was well-validated and can be regarded as a reliable representation of the normal spine.

### Biomechanical change with different extents of facetectomy

**Fig. 5:**
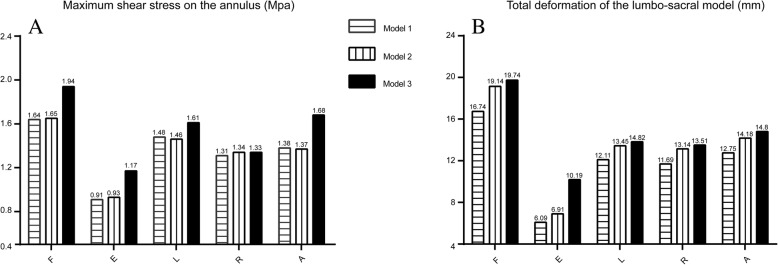
Biomechanical changes in three models. **a**. maximum shear stress on the annulus in L5-S1 disc. **b**. Total deformation of the lumbo-sacral model. Model1 The complete model. Model2 The model after one-quarter excision of the superior articular process. Model3 The model after one-half excision of the superior articular process. F. Flexion. E. Extension. L. Left lateral bending. R. Right lateral bending. A. Axial rotation

We choose the L5-S1 segment IVD to investigate changes in maximum shear stress on the annulus due to the elevated levels of morbidity associated with LDH of this segment.The maximum annulus shear stress in the L5 IVD increased with larger extents of facetectomy under most conditions, except during right lateral bending. The variation of the total deformation shows the same trend and which was most significant under extension in model 3, as it increased by 67.3 and 47.5%, compared to models 1 and 2, respectively (Fig. 5).

**Fig. 6:**
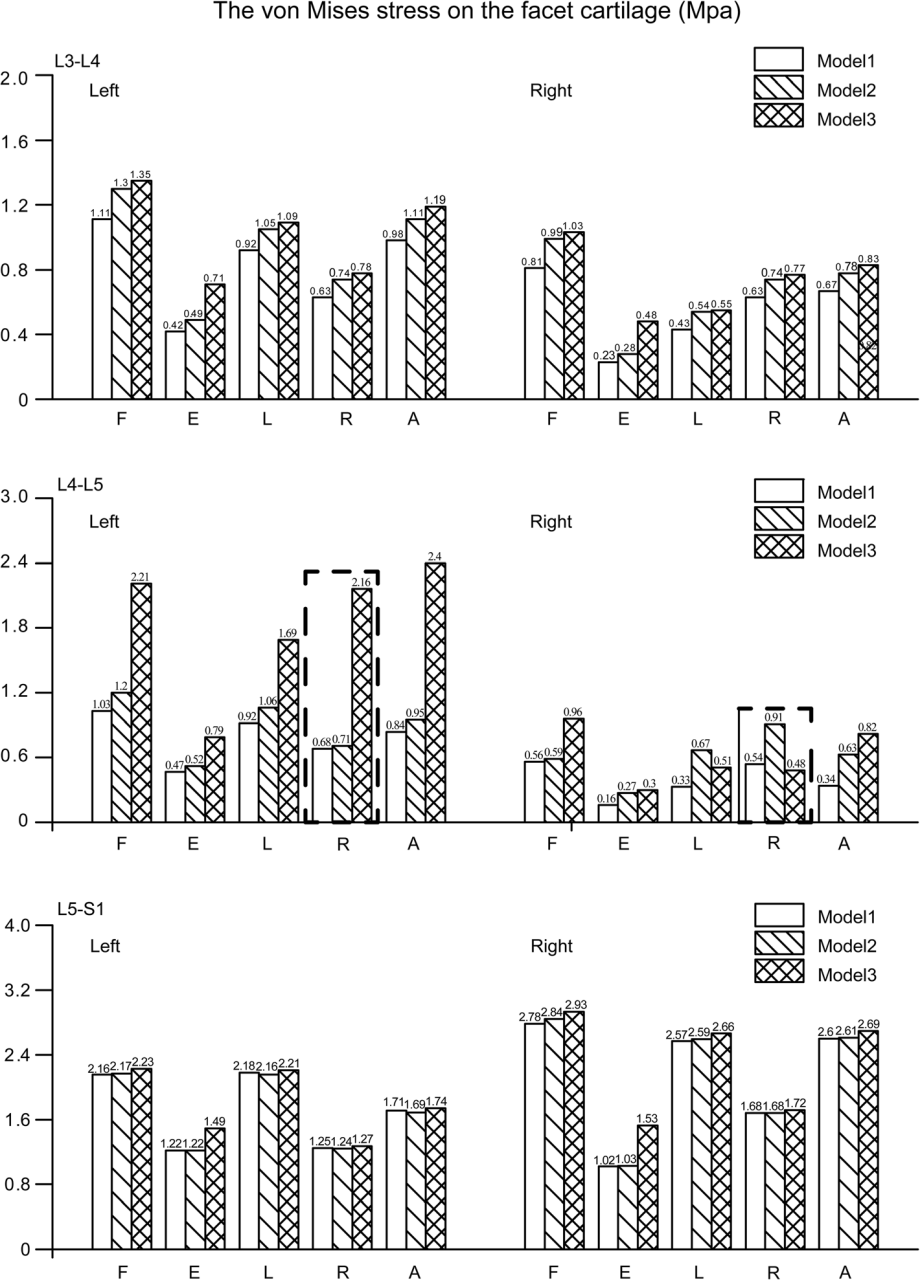
von Mises stress on the facet cartilage. Model1 The complete model. Model2 The model after one-quarter excision of the superior articular process. Model3 The model after one-half excision of the superior articular process. F. Flexion. E. Extension. L. Left lateral bending. R. Right lateral bending. A. Axial rotation

**Fig. 7:**
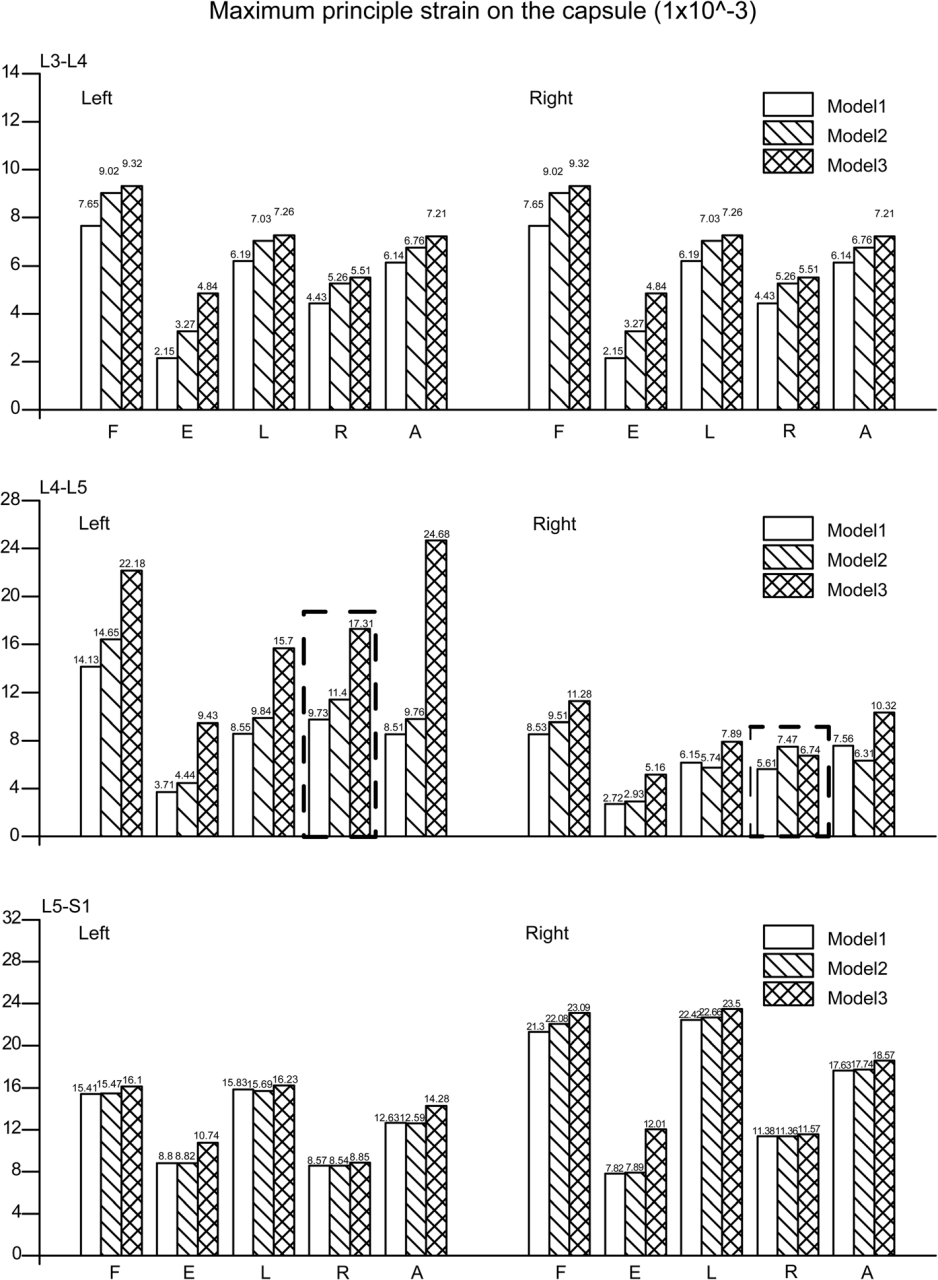
Maximum principle strain on the capsule. Model1 The complete model. Model2 The model after one-quarter excision of the superior articular process. Model3 The model after one-half excision of the superior articular process. L left side. R right side. F. Flexion. E. Extension. L. Left lateral bending. R. Right lateral bending. A. Axial rotation

The variation in maximum principle strain on the capsule and von Mises stress on the facet cartilage in the L3–L4 segment showed a similar trend, as values increased with higher grades of facetectomy under all loading conditions. With respect to the operated segment (L4–L5), an exception was observed during right lateral bending, in which higher grades of facetectomy led to lower maximum principle strain on the capsule and lower von Mises stress on the facet cartilage in the operated side of the facet joint. Interestingly, corresponding values in the opposite side under the same loading conditions were dramatically higher; compared to the values in models 1 and 2, the maximum principal strain in model 3 increased by 77.9 and 51.8% and von Mises stress in the facet cartilage increased by 217.6 and 204.2%, respectively. The patterns of variation in the L5–S1 segment were consistent with those of the upper two segments, but note that all values are obviously higher in this segment. The patterns of change in the two values under different loading conditions are shown in Figs. 6 and 7.

## Discussion

FBSS is a significant postoperative complication with typical symptoms of persistent LBP and has been reported to be an important predictor of postoperative poor outcome [1, 22, 36]. Considering that biomechanical deterioration is the key trigger of postoperative complications [2, 4–6], the investigation of the biomechanical conditions of FBSS is vital to reduce its risk.

Compared to traditional open discectomy, PTED may reduce the risk of FBSS. Previously studies reported that open posterior lumbar surgery will lead to the damage and atrophy of paraspinal muscles, which have been indicated as a significant cause of FBSS [36–38]. Meanwhile, the weakness of paraspinal muscle will result in lumbar instability, a significant trigger of FBSS [1, 39]. As a minimally invasive surgical method, PTED will not damage the paraspinal muscles and the conclusion that PTED reduces the risk of FBSS seems credible [39, 40]. However, clinical studies have shown that minimally invasive surgical methods do not significantly decrease morbidity associated with postoperative complications compared to open surgery, and the damage to osteoligamentous structures during the microdiscectomy procedure can also result in biomechanical deterioration [5, 37, 41]. Thus, the conclusion that minimally invasive spine surgery could decrease the risk of FBSS may be unreliable. Given these contradictory conclusions, it is critical to pay attention on the relationship between PTED and FBSS from a biomechanical point of view.

Facetectomy is needed in PTED for accessing the intraspinal region. For patients who suffer from hypertrophy of the facet joints, larger extents of facetectomy are important for exiting nerve root decompression. However, such procedure may be closely associated with postoperative complications [6, 9, 11, 42].

To identify the effect of various extents of facetectomy on morbidity during FBSS, an issue that has not been clearly studied, a three-dimensional lumbosacral model was reconstructed and validated to assess changes in biomechanical indexes that are directly associated with FBSS, namely, maximum shear stress on the annulus in the L5 segment, von Mises stress at the facet cartilage, maximum principle capsular strain on the capsule and the total deformation of current models.

Causes of LBP that involve facet joints have been recognised as risk factors for more than half century [19, 43], and changes in the force applied on the facet surface and to capsular strain are closely associated with facet joint diseases [18, 23, 44]. In the current study, we have discovered that von Mises stress on the facet cartilage increases with larger extents of facetectomy, which may accentuate degeneration of the facet joints [45, 46], while an increase in the maximum principal strain on the capsule can result in local neuropathic pain [16, 17]. Thus, our results indicate that larger extents of facetectomy may lead to facet joint diseases and LBP, especially in the contralateral side and when the body bends in the direction of the operated side.

Notably, we show that the stress on the facet cartilage and the strain on the capsule were the highest in the L5–S1 segment, and such phenomenon may occur when biomechanical indexes is applied close to a fixed surface and specific data for this segment may be less reliable. Nonetheless, as this is a qualitative rather than a quantitative study, we believe that the results are credible.

**Fig. 8:**
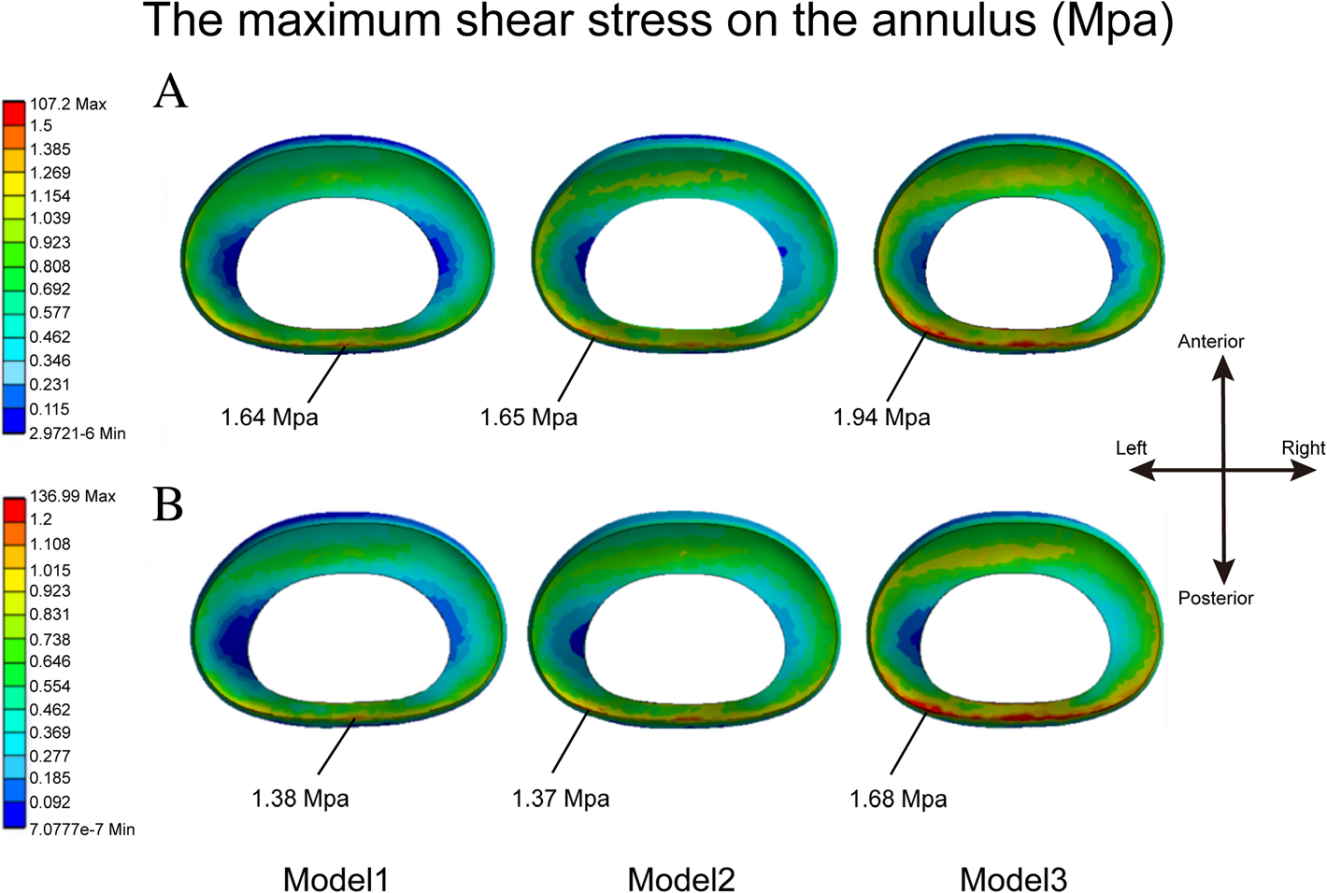
The nephogram of shear stress on the annulus in L5 disc. **a**. In flexion. **b**. In axial rotation. Model1 The complete model. Model2 The model after one-quarter excision of the superior articular process. Model3 The model after one-half excision of the superior articular process

An increase in the maximum shear stress on the annulus is an important factor that results in radical annulus tears, which may lead to discogenic LBP and LDH, two key triggers of FBSS [1, 12, 25, 26, 36]. In this study, the maximum shear stress on the annulus obviously increased during flexion and axial rotation with larger extents of facetectomy, and stress concentration can be observed at the posterior annulus (Fig. 8). In addition, the variation trends of biomechanical indexes in the annulus and the facets are same. Considering that facet cartilages and capsules will protect the posterior annulus during torsion and flexion, respectively [15], the risk of annulus tears may be increased with larger extents of facetectomy.

Facet joints are crucial for spine stabilisation, and spine instability is a common cause of FBSS [21, 22, 47, 48]. Postoperative hypermobility will accelerate disc degeneration and result in FBSS [49, 50]. The increase in the total deformation of the current models is a good indicator of lumbar instability, and we observed this phenomenon with larger extents of facetectomy under all loading conditions.

Finally, note that some patients suffer from severe postoperative LBP even if typical changes in imaging are absent [1, 51]. In such patients, we hypothesise that postoperative deterioration of biomechanical indexes leads to LBP before an organic change could be detected by imaging. Importantly, this phenomenon is further supported by the necessity to avoid, or at least reduce, biomechanical deterioration by surgical intervention. In summary, the results from this study suggest that less facetectomy is better in PTED on the premise of achieving the goal surgery, i.e. to reduce morbidity due to FBSS.

The current conclusion must be accepted on the premise of the awareness of the following limitations. LDH patients who underwent PTED present with varying grades of disc degeneration, facet degeneration and osteoporosis. In addition, this study was conducted under a single load condition rather than different load conditions. These factors influence the change in biomechanical conditions of the spine after facetectomy and can thereby affect risk in FBSS [13, 17, 25, 44]. However, these factors were not incorporated into the present study, and we expect to analyse their effects in future studies.

## Conclusions

Less facetectomy is better in PTED for which may reduce the risk of biomechanical deterioration and consequently FBSS.

## Data Availability

All the data of the manuscript are presented in the paper.

## Abbreviations

CT: Computed tomography
FBSS: Failed back surgery syndrome
FEM: Finite element methods
IVD: Intervertebral disc
LBP: Low back pain
LDH: Lumbar disc herniation
LF: Ligamentum flavum
PTED: Percutaneous transforaminal endoscopic discectomy
